# The interrelations of radiologic findings and mechanical ventilation in community acquired pneumonia patients admitted to the intensive care unit: a multicentre retrospective study

**DOI:** 10.1186/1476-0711-13-5

**Published:** 2014-01-08

**Authors:** Hakan Erdem, Zeliha Kocak-Tufan, Omer Yilmaz, Zuhal Karakurt, Aykut Cilli, Hulya Turkan, Ozlem Yazicioglu-Mocin, Nalan Adıguzel, Gokay Gungor, Canturk Taşcı, Gulden Yilmaz, Oral Oncul, Aygul Dogan-Celik, Ozcan Erdemli, Nefise Oztoprak, Yakup Tomak, Asuman Inan, Demet Tok, Sibel Temur, Hafize Oksuz, Ozgur Senturk, Unase Buyukkocak, Fatma Yilmaz-Karadag, Derya Ozturk-Engin, Dilek Ozcengiz, Ahmet Karakas, Hayati Bilgic, Hakan Leblebicioglu

**Affiliations:** 1 Department of Infectious Diseases and Clinical Microbiology, GATA Haydarpasa Training Hospital, Istanbul, Turkey; 2 Department of Infectious Diseases and Clinical Microbiology, Ankara Atatürk Training & Research Hospital, Yildirim Beyazit University, Bilkent Ankara, Turkey; 3 Department of Radiology, School of Medicine, Suleyman Demirel University, Isparta, Turkey; 4 Sureyyapasa Chest Diseases and Thoracic Surgery Education and Research Hospital, Respiratory Intensive Care Unit, Istanbul, Turkey; 5 Department of Pulmonary Diseases, School of Medicine, Antalya, Turkey; 6 Department of Anesthesiology and Reanimation, Gulhane Medical Academy, Ankara, Turkey; 7 Department of Pulmonary Diseases, Gulhane Medical Academy, Ankara, Turkey; 8 Department of Infectious Diseases and Clinical Microbiology, Ankara University School of Medicine, Ankara, Turkey; 9 Department of Infectious Diseases and Clinical Microbiology, School of Medicine, Trakya University, Edirne, Turkey; 10 Department of Anesthesiology and Reanimation, Yuksek Ihtisas Training and Research Hospital, Ankara, Turkey; 11 Department of Infectious Diseases and Clinical Microbiology, Antalya Training and Research Hospital, Antalya, Turkey; 12 Department of Anesthesiology and Reanimation, School of Medicine, Sakarya University, Sakarya, Turkey; 13 Department of Infectious Diseases and Clinical Microbiology, Haydarpasa Numune Training and Research Hospital, Istanbul, Turkey; 14 Department of Anesthesiology and Reanimation, School of Medicine, Celal Bayar University, Manisa, Turkey; 15 Department of Anesthesiology and Reanimation, School of Medicine, Yeditepe University, Istanbul, Turkey; 16 Department of Anesthesiology and Reanimation, School of Medicine, Sutcu Imam University, Kahramanmaras, Turkey; 17 Department of Anesthesiology and Reanimation, School of Medicine, Maltepe University, Istanbul, Turkey; 18 Department of Anesthesiology and Reanimation, School of Medicine, Kirikkale University, Kirikkale, Turkey; 19 Department of Infectious Diseases and Clinical Microbiology, Goztepe Training and Research Hospital, Medeniyet University, Istanbul, Turkey; 20 Department of Anesthesiology and Reanimation, School of Medicine, Cukurova University, Adana, Turkey; 21 Department of Infectious Diseases and Clinical Microbiology, Gulhane Mecial Academy, Ankara, Turkey; 22 Department of Infectious Diseases and Clinical Microbiology, School of Medicine, Ondokuz Mayis Iniveristy, Samsun, Turkey

**Keywords:** Radiography, Thoracic, Pneumoniae, Imaging, Critical care

## Abstract

**Background:**

We evaluated patients admitted to the intensive care units with the diagnosis of community acquired pneumonia (CAP) regarding initial radiographic findings.

**Methods:**

A multicenter retrospective study was held. Chest x ray (CXR) and computerized tomography (CT) findings and also their associations with the need of ventilator support were evaluated.

**Results:**

A total of 388 patients were enrolled. Consolidation was the main finding on CXR (89%) and CT (80%) examinations. Of all, 45% had multi-lobar involvement. Bilateral involvement was found in 40% and 44% on CXR and CT respectively. Abscesses and cavitations were rarely found. The highest correlation between CT and CXR findings was observed for interstitial involvement. More than 80% of patients needed ventilator support. Noninvasive mechanical ventilation (NIV) requirement was seen to be more common in those with multi-lobar involvement on CXR as 2.4-fold and consolidation on CT as 47-fold compared with those who do not have these findings. Invasive mechanical ventilation (IMV) need increased 8-fold in patients with multi-lobar involvement on CT.

**Conclusion:**

CXR and CT findings correlate up to a limit in terms of interstitial involvement but not in high percentages in other findings. CAP patients who are admitted to the ICU are severe cases frequently requiring ventilator support. Initial CT and CXR findings may indicate the need for ventilator support, but the assumed ongoing real practice is important and the value of radiologic evaluation beyond clinical findings to predict the mechanical ventilation need is subject for further evaluation with large patient series.

## Background

The first line therapy of CAP is usually managed outside the hospital. However, in case of a failure in the first line antibiotherapy or in patients stratified as severe according to the severity indexes, hospital admission has to be considered. Although the critical initial investigation is CXR to check complications, further imaging with computerized tomography (CT) or ultrasound (US) examinations will be needed in case of a suspicion for empyema or abscess [1,2]. ICU admission is another issue and according to the guidelines and findings reflecting acute respiratory failure, severe sepsis or septic shock and radiographic extension of infiltrates, as well as severely decompensated co-morbidities, should prompt consideration of admission to the ICU or an intermediate care unit. In the ICU admission algorithm of the current guidelines, mechanical ventilation is one of the major criteria along with septic shock [3-5].

A large multicenter study was held to evaluate the mortality indicators on the clinical grounds for the patients who were admitted to the ICUs with the diagnosis of CAP, and discussed elsewhere [6]. We suggest that the presentation of CAP which ends with an ICU admission and requirement of mechanical ventilation may have different patterns in terms of radiographic findings. In this study, we aimed to evaluate those patients regarding initial radiographic findings and aimed to explore the correlations, if exist, of images with the need for mechanical ventilation.

## Methods

### Database of the study

The above mentioned main study was a 19-centers retrospective study on the CAP patients admitted to the ICUs (Erdem H, Hulya Turkan, Aykut Cilli, et al. Mortality indicators in community-acquired pneumonia requiring intensive care in Turkey. International Journal of Infectious Diseases, in press). The database of that study was used in this study to evaluate the radiological findings detected with CXR and CT. Informed consent was not required, and the study was approved by Local Ethics Committee.

### Patients

Data were collected from all patients admitted to the participant ICUs with the diagnosis of CAP thorough October 2008 to January 2011. Patients with pulmonary tuberculosis were excluded. The enrolled patients were consecutive cases aged ≥16 years, with conclusive evidence of CAP as primary diagnosis, confirmed by CXR: Pneumonia was defined as the presentation of acute onset of symptoms suggestive of lower respiratory tract infection and radiographic evidence of a new infiltrate [3-5].

Patients were admitted to the ICU either because they required mechanical ventilation or because they were judged to be in an unstable condition requiring intensive care. The patients were evaluated for their radiological findings and the eligible ones were included into this study, whoever with inconclusive or indeterminate findings on CXR was excluded. Lack of information on detailed findings of the images (consolidation, abscess, interstitial patch and cavitations) in documents which were fulfilled by the cooperating centers was defined as indeterminate or inconclusive findings.

When the correlation analysis were made between the mechanical ventilation need and the radiological findings, only those who were only on noninvasive mechanical ventilation (NIV) or only on invasive mechanical ventilation (IMV) were compared, those who needed NIV with subsequent IMV or vice versa were excluded from the statistics.

### Design and variables

CT and CXR variables include 1) the definition of the findings: consolidation, interstitial/patchy involvement, abscess and cavitations; and also 2) the localization: unilateral multi-lobar (more than two lobes) involvement and bilateral involvement.

### Statistical analysis

SPSS for windows 14.0 (SPSS Inc, Chicago; IL) was used for statistical analysis. Variables are described as meanÂ + -standard deviation (SD) or percentages. Clinical significance of radiological finding were analyzed with chi-square test and when a cell have expected count less than five with Fishers’ exact test. Odds ratio (OR) and 95% confidence intervals (CIs) were given for each significant radiological variable. For the calculation of correlations between CXR and CT findings, kappa statistics were used. In multivariate analyses, all of the radiological parameters were included due to their clinical significance.

## Results

From 445 patients 57 patients were excluded because of inconclusive or indeterminate findings. A total of 388 patients were enrolled. Of all, 265 (68%) were males and 123 (32%) were females; the mean age was 62Â + - 18.2 years (min 16, max 95). Consolidation was the main finding on CXR (89.2%) and CT (79.7%) examinations. Of all, 44.7% and 44.9% had multi-lobar involvement on CXR and CT, respectively. Bilateral involvement was found in 39.3% on CXR and 43.5% on CT. Abscesses and cavitations were rarely found. Radiological findings of the patients were shown in detail on Table 1.

**Table 1:** Chest X-ray and computerized tomography findings of the patients

**n (%)**	**X-ray (n=388)**	**CT (n=69)**
**Consolidation**	346 (89.2%)	55 (79.7%)
**Interstitial/patchy involvement**	105 (27.1)	31 (44.9%)
**Abscess**	5 (1.3%)	1* (1.4%)
**Cavitation**	8 (2.1%)	0**
**Involvement >2 lobe**	155 (44.7%)	31 (44.9%)
**Bilateral involvement**	152 (39.3%)	30 (43.5%)

*Only one of five patients with abscess on CXR had CT evaluation, remaining four had not. CT was also positive for abscess for that one patient. **None of the patients with cavitation on CXR had CT evaluation, so comparison or confirmation with CT was unavailable for cavitation.

From all 388 patients, 69 patients (18%) did not need any kind of ventilator sport while a total of 319 patients (82%) needed mechanical ventilation: 167 (43%) patients required IMV and 221 (57%) patients needed NIV. From the 319 patients who needed ventilator support 69 patients needed subsequent uses of both IMV and NIV and excluded from the statistical calculations to not confuse the results. So, statistical analysis for comparison of IMV and NIV were performed among the remaining 250 patients who just needed IMV or NIV: 152 patients who required NIV and 98 patients who needed IMV. In univariate analysis, although NIV requirement was seen to be more common in patients with multilobar involvement on CXR comparing with those who didn’t have multilobar involvement, the difference was statistically insignificant (p = 0.1). IMV need was common in those patients with cavity on CXR (p = 0.03) while NIV requirement was remarkable in patients with consolidation on CT (p < 0.001).

Radiological findings with respect to IMV and NIV are shown in Table 2.

**Table 2:** Radiological findings with respect to invasive and noninvasive mechanical ventilation

**Radiological findings**	**NIV**			**IMV**		
	**n**	**%**	**p**	**n**	**%**	**p**
**Chest X Ray**	**(n=152)**				**(n=98)**	
Consolidation	135	88.8	ns	89	90.8	ns
Interstitial involvement	40	26.3	ns	23	23.5	ns
Cavity	1	0.7	ns	5	5.1	**0.03**
Abscess	0	-	-	2	2.1	ns
Multilobar involvement	59	42.4	ns	45	54.2	ns
Bilateral involvement	63	41.4	ns	42	42.9	ns
**Computerized tomography**	**(n=21)**			**(n=19)**		
Consolidation	10	47.6	**<0.001**	16	84.2	ns
Interstitial involvement	12	57.1	ns	8	42.1	ns
Cavity	0	-	-	0	-	-
Abscess	0	-	-	0	-	-
Multilobar involvement	6	28.6	ns	6	31.6	ns
Bilateral involvement	8	38.1	ns	11	57.9	ns

ns: not significant.

In multivariate analyzes, among all CXR findings, only multilobar involvement on CXR was found to be a risk factor for NIV (OR: 2.36, 95% CIs: 1.27-4.38, p = 0.006). No relation was found between CXR findings and IMV. Regarding CT findings, consolidation on CT was a risk factor for NIV (OR: 47.49,%95CIs:3.77-597.56, p = 0.03) and multilobar involvement was a risk factor for IMV (OR: 7.77 95% CIs:1.26-47.94, p = 0.027). If we would take the p value as 0.1, interstitial involvement on CT could be a risk factor for NIV as well (OR: 6.33, 95% CIs: 0.66-61.23 p = 0.1). None of the patients with cavitations on CXR had CT evaluation. Thus, analysis for the cavitations and mechanical ventilation could not be performed.

### CT vs CXR

Of all, there were 69 patients who had both CT and CXR examinations available. The highest correlation between CT and CXR findings was observed for interstitial involvement (κ = 0.50, p < 0.001). Correlation of consolidation between two imaging modality was also significant (κ = 0.32, p = 0.003). Only one of five patients who had abscesses on CXR had also CT examination and this patient’s CT images also have disclosed abscess. None of the patients who were reported to have cavitations in CXR had CT; thereby correlation analysis could not be performed for abscess and cavitations. Although statistically significant, correlations between CXR and CT were weak for bilateral and multilobar involvement (κ =0.22, p = 0.05 and κ =0.35, p = 0.003).

## Discussion

The radiographic findings and their interrelations with mechanical ventilation for CAP patients admitted to the ICUs were evaluated in this study. Although studies has shown that CXR has only around 30% specificity on diagnosis of pneumonia, still CXR is the key tool with its excellent cost-benefit ratio in the diagnosis, especially if it demonstrates a characteristic area of alveolar consolidation [7]. The sensitivity and specificity of clinical findings on physical examination were reported to be 58% and 67%, respectively [1]. When the radiography is negative, the significance of the clinical findings is unclear [4].

The initial images of CXR may not disclose radiological findings in one-fifth of CAP patients. However, in more than half of these patients without initial radiological confirmation, infiltrates had been observed on subsequent CXR [8]. CT is also needed in some cases to clear out the diagnosis, particularly in case of complications. Since the inclusion criteria of our group was mainly dependent on radiographic findings, at least on CXR, the comparison between the radiology negative and positive groups were unavailable in our study.

The main radiographic finding of our CAP patients requiring intensive care was consolidation (89% in CXR and 80% in CT); interstitial involvement was found in one-fourth of the cases in CXR and in approximately half of CT examinations. Abscesses and cavitations were rare findings. Advanced findings like multilobar and bilateral involvements were also common findings seen in up to half in our study group.

In the routine clinical work, CXR is usually evaluated by the physician while the CT is almost all the time assessed by the radiologist. Thus, physicians and radiologists are frequently known to disagree whether a patient has pneumonia or not. In one study, nearly half of patients with the diagnosis of pneumonia by the physician were found to be normal or have other diseases than pneumonia by the radiologist [9]. The correlation between CT and CXR findings in our study was highest for interstitial involvement in half followed by multilobar involvement and consolidation in one-third and bilateral involvement in one-fifth. Although correlations to a degree seem to exist between CXR and CT in terms of main radiological variables, CT with better resolution should be considered in the diagnosis of pulmonary disorders undetected by CXR. Thus, CT should be taken into consideration when initial portable chest roentgenogram and clinical course of the ICU patient with CAP do not correlate.

Unfortunately, only 18% of our study group had CT evaluation. This is probably due to the difficulties in performing chest CT for those unstable or for patients with severe respiratory failure hospitalized in the ICU. Therefore, comparison of all findings on CXR and CT were unlikely. When we consider older age (mean age 62) and severity of our cases one can expect a higher CT use but only one of six cases underwent CT evaluation. Hence, we may also suggest that although available in all centers CT is not routinely used for all patients with suspected CAP in daily practice, even in severe cases like our subjects.

A big number of our patients needed ventilator support. NIV requirement was seen to be more common in those with multilobar involvement on CXR as 2.4-fold and consolidation on CT as 47-fold compared with those who do not have these findings. On the other hand, IMV need increased 8-fold in patients with multilobar involvement on CT. No association was found between CXR findings and IMV. Therefore, we suggest that patients with consolidation and multilobar involvement require a very close follow up for the subsequent need for mechanical ventilation.

In general medical practice, the usual CAP is so called-lobar pneumonia and it is usually limited to one lobe or segment [10]. Even though the multilobar infiltrates are of minor criteria and cavitation is not included in the list for admission of the CAP patients to the ICU [3-5], they seem to deserve major attention according to our findings. Our results confirm that the detection of multilobar involvement and the presence of consolidation would frequently require mechanical ventilation in CAP patients in the ICUs. On the other hand, our study could not involve the subsequent images of our patients. It was shown that radiologic progression of pulmonary infiltrates within the first 48 hours in patients with severe CAP is an independent predictor of adverse outcome with a threefold increase in the risk of death [11]. However, some other studies do not support the sequential CXR since it is believed not to have a significant impact on clinical management of the disease [12,13]. Thus our data should be interpreted as the efficacy of initial imaging during ICU admission in predicting the need of mechanical ventilation.

In conclusion CAP patients who are admitted to the ICU are severe cases requiring ventilator support. CXR and CT findings correlate up to a limit in terms of interstitial involvement and consolidation as well as bilateral and multilobar involvement. CXR is used widely and CT evaluation is not available for all patients. Although the correlations were not very high between two modalities, both can be used in daily practice. We found a certain relation between mechanical ventilation need and radiological findings, but the assumed ongoing real practice is important and the value of radiologic evaluation beyond clinical findings to predict the mechanical ventilation need is subject for further evaluation with large patient series.

## Abbreviations

CAP: Community acquired pneumoniae; CXR: Chest X ray; CT: Computerized tomography; ICU: Intensive care unit; IMV: Invasive mechanical ventilation; NIV: Noninvasive mechanical ventilation.

## Competing interests

The authors declare that they have no competing interest.

## Authors’ contributions

HE: Study supervision, study concept and design, acquisition of the data, critical revision of the manuscript for important intellectual content. ZKT: Analysis and interpretation of the data, drafting of the manuscript, statistical expertise. OY: Drafting of the manuscript, critical revision of the manuscript for important intellectual content. Other authors: Critical revision of the manuscript for important intellectual content and acquisition of the data. All authors declare that they have no competing interest. All authors read and approved the final manuscript.
